# The effects of water control on the survival and growth of *Alternanthera philoxeroides* in the vegetative reproduction and seedling stages

**DOI:** 10.1038/s41598-021-92674-2

**Published:** 2021-06-30

**Authors:** Xuemei Peng, Yunfei Yang, Xiumin Yan, Haiyan Li

**Affiliations:** 1 School of Biological Sciences, Guizhou Education University, Guiyang, 550018 People’s Republic of China; 2 Key Laboratory of Vegetation Ecology, Ministry of Education, Institute of Grassland Science, Northeast Normal University, Changchun, 130024 People’s Republic of China; 3 School of Geography and Resources, Guizhou Education University, Guiyang, 550018 People’s Republic of China

**Keywords:** Invasive species, Population dynamics, Ecology

## Abstract

*Alternanthera philoxeroides* (Martius) is an infamous invasive alien plant that is widely distributed in aquatic and terrestrial habitats. To investigate the vegetative reproduction, growth, survival strategy, and the function of leaves in fragment of *A. philoxeroides* under different water conditions, two water control experiments were conducted with different leaf treatments: (1) water control with stolon fragments, and (2) water control with plants. The water control was subjected to five levels: I 30% soil water content, II 70% soil water content, III 97% soil water content, IV water depth of 5 cm, and V water depth of 10 cm in combination with the two leaf treatments, fragments with two leaves and fragments without leaves. Based on the results, *A. philoxeroides* produced a significantly higher stem length, node number, leaf number, stem biomass, leaf biomass, and total biomass in the 97% soil water content and in treatments with leaves. Additionally, the stem mass ratio increased and the root mass ratio decreased with the increase of the water content. In Exp. 1, the survival rate was the highest in the 97% water content and was 0 in the 30% water content. Therefore, the leaves of stolon fragments contribute to the vegetative reproduction and growth of *A. philoxeroides*. In response to different water conditions, *A. philoxeroides* adopts different strategies according to the resource reserves by itself, which are conducive to its survival and widespread occurrence.

## Introduction

The reason for the successful invasion of alien invasive plants in their introduced ranges is a primary question and hot topic in invasion biology^1,2^. *Alternanthera philoxeroides* (Martius) is a notorious invasive weed, which can successfully invade diverse habitats with considerably varying degrees of water availability (from dry terrestrial to aquatic habitats), and it is widespread throughout the world^3^. Its invasion seriously endangers the diversity of native plants in many parts of the world^4,5^. Studies have shown that *A. philoxeroides* has lower genetic diversity^6,7^, higher phenotypic plasticity^8,9^, a higher growth rate^10^, more vegetative reproduction^11^, and a stronger ability to spread than its native congener species^10,12^. It also has different anatomical structures in different water conditions^13^.

Submergence is common in the distribution range of *A. philoxeroides*^14^. Submersion experiments have been carried out on the plants of *A. philoxeroides*, and the results showed that plant survival was 100% during 2 weeks of complete submergence at various water depths^15^. However, submergence negatively affected the growth of *A. philoxeroides*; for example, the stem length, stem diameter, node number, leaf number, total biomass, root biomass allocation, and so on decreased under sumbergence^15,16^. In addition, clonal integration could significantly increase growth and clonal reproduction of the apical ramets and contributed greatly to the apical ramets of *A. philoxeroides* subjected to submergence^14^. Most of the previous studies only considered the environmental water conditions^17–19^, but did not combine the conditions of the plants themselves (such as the developmental stage of the plant) in their investigations. Therefore, investigation on the growth performance of a plant in the vegetative reproduction and seedling stages in response to water availability will be helpful to elucidate the strategies adopted by *A. philoxeroides* to cope with different water conditions.

In addition to being the site of photosynthesis, leaves can also store carbohydrates and proteins^20–22^. After the fragmentation of *A. philoxeroides* stems, leaf storage increases the survival rate and growth (biomass, leaf area, ramet number, stem length, and leaf number) of new ramets^23^. However, the strong transpiration of leaves can carry water away from the plant^24^. When the stem is fragmented, the transpiration of leaves accelerates the loss of water in leaves and stem fragments. Whether this inhibits the vegetative reproduction or not and whether different water conditions change the effect of leaf transpiration on vegetative stolon fragments have not been answered by current studies.

We propose to use stolon fragments with or without leaves to carry out water control experiments at five levels on vegetative reproduction fragments and the plants half a month after the reproduction of *A. philoxeroides* to address the following questions: (1) Can stolon fragments of *A. philoxeroides* reproduce asexually under drought and submergence? (2) Under what water conditions can *A. philoxeroides* survive? (3) What strategies do *A. philoxeroides* adopt under different water conditions? (4) What is the effect of leaves on the fragment of *A. philoxeroides* under different water conditions?

## Results

### Water control with stolon fragments (Exp. 1)

**Figure 1:**
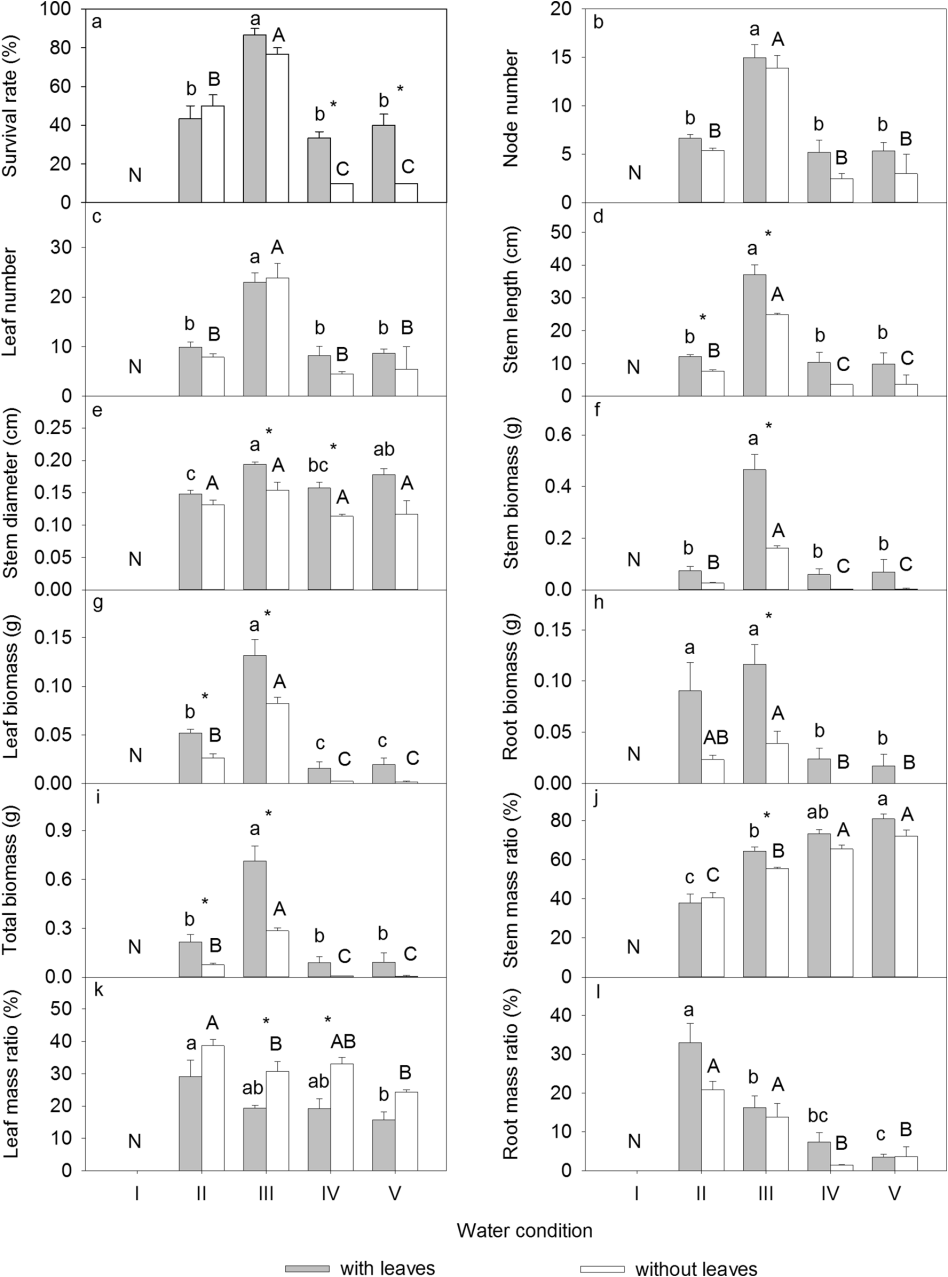
Survival rate, morphological traits, biomass, and biomass allocation of *A. philoxeroides* in Exp. 1. Means + SE are given. I, 30% soil water content; II, 70% soil water content; III, 97% soil water content; IV, water depth of 5 cm; V, water depth of 10 cm. Different uppercase and lowercase letters indicate significant differences among different water conditions. *indicates significant differences between different leaf treatments. An uppercase N indicates no data.

The water conditions and leaf treatments affected all measurements of *A. philoxeroides* plants in Exp. 1 (Table 1). The survival rate under the 97% water content was significantly higher than that under the other water condition treatments (*P* < 0.05); the fragments did not sprout under the 30% water content (Fig. 1a). The node number, leaf number, stem length, stem diameter, stem biomass, leaf biomass, root biomass, and total biomass under the 97% water content were significantly larger than those under the other water condition treatments (Fig. 1b-i) (*P* < 0.05). The stem mass ratio increased significantly with an increasing water level, while the leaf mass ratio and root mass ratio decreased significantly with an increasing water level (Fig. 1j, k, l) (*P* < 0.05). The measured values were larger for fragments with leaves, except for the leaf mass ratio (Fig. 1).

**Table 1:** Two-way ANOVA for the survival rate, morphological traits, biomass, and biomass allocation of *A. philoxeroides* in Exp. 1. The degree of freedom (DF), F values and significance levels (*** *P* < 0.001, ** *P* < 0.01, * *P* < 0.05) are given.

	Leaf treatment	Water condition	Leaf treatment × Water condition
DF	1	3	3
Survival rate	17.291***	67.815***	5.753**
Node number	5.657*	43.632***	0.268
Leaf number	1.990	34.526***	0.537
Stem length	20.268***	53.786***	1.187
Stem diameter	35.669***	7.059**	1.822
Stem biomass	25.263***	37.241***	7.637**
Leaf biomass	20.902***	65.119***	1.995
Root biomass	17.239***	9.363**	1.861
Total biomass	27.123***	38.816***	5.869**
Stem mass ratio	8.052*	66.410***	2.105
Leaf mass ratio	25.589***	7.415**	0.256
Root mass ratio	5.438*	25.767***	1.553

### Water control with plants (Exp. 2)

**Figure 2:**
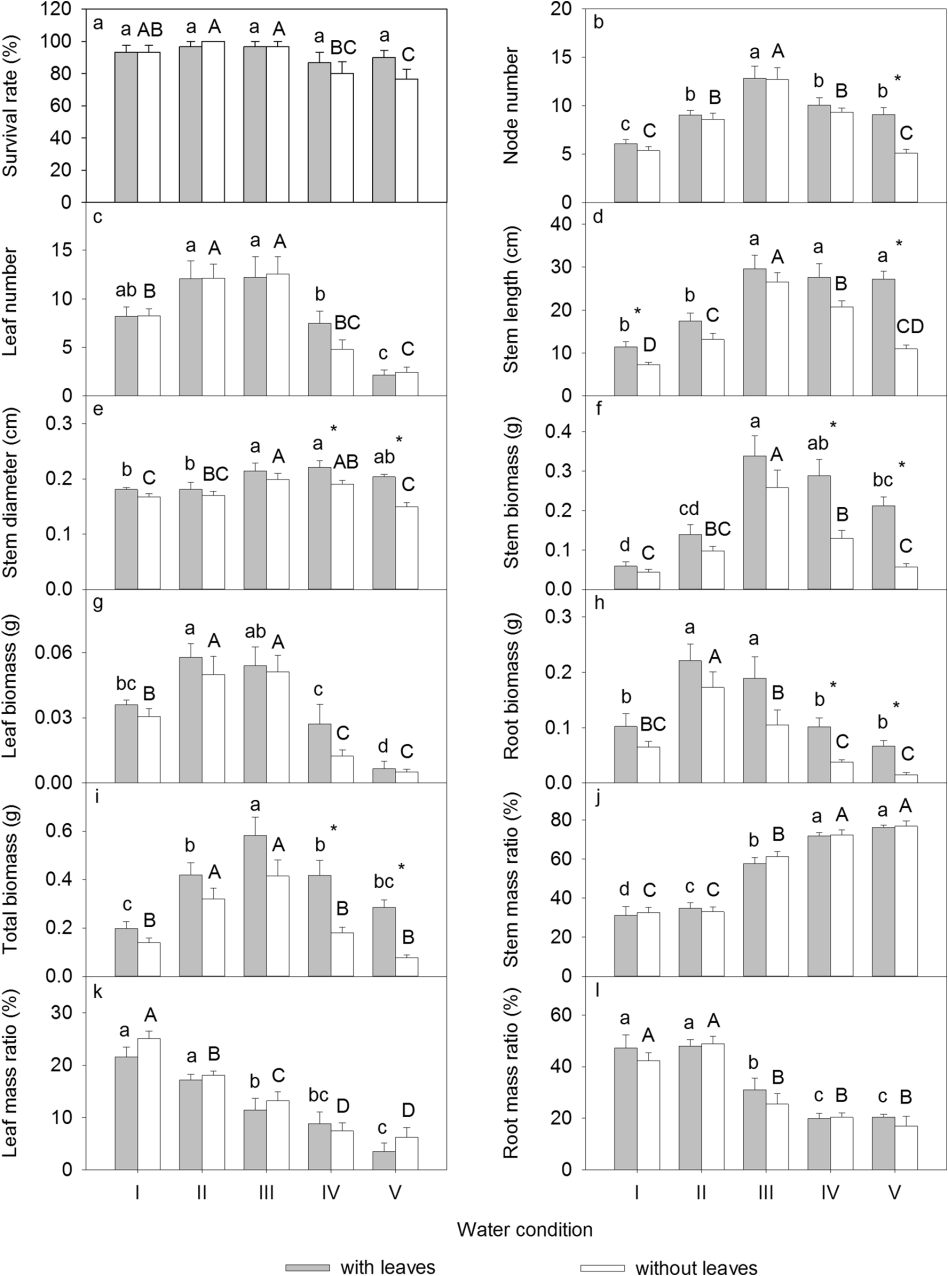
Survival rate, morphological traits, biomass, and biomass allocation of *A. philoxeroides* in Exp. 2. Means + SE are given. I, 30% soil water content; II, 70% soil water content; III, 97% soil water content; IV, water depth of 5 cm; V, water depth of 10 cm. Different uppercase and lowercase letters indicate significant differences among different water conditions. *indicates significant differences between different leaf treatments.

The water conditions and leaf treatments affected all measurements of *A. philoxeroides* plants in Exp. 2 (Table 2). There was no significant difference in the survival rate between the water conditions for treatments with leaves, while the survival rate was significantly decreased under submergence for treatments without leaves (Fig. 2a). The node number, leaf number, stem length, stem diameter, stem biomass, leaf biomass, root biomass, and total biomass under the 97% water content were significantly higher than those under the other water conditions (Fig. 2b-i) (*P* < 0.05). The stem mass ratio increased significantly with an increasing water level, while the leaf mass ratio and root mass ratio decreased significantly with an increasing water level (Fig. 2j-l) (*P* < 0.05). The measured values were higher for treatments with leaves, except for the leaf mass ratio (Fig. 2).

**Table 2:** Two-way ANOVA for the survival rate, morphological traits, biomass, and biomass allocation of *A. philoxeroides* in Exp. 2. The degree of freedom (DF), F values and significance levels (*** *P* < 0.001, ** *P* < 0.01, * *P* < 0.05) are given.

	Leaf treatment	Water condition	Leaf treatment × Water condition
DF	1	4	4
Survival rate	1.238	4.653**	0.990
Node number	6.559*	26.424***	2.247
Leaf number	0.221	20.173***	0.461
Stem length	31.410***	28.229***	3.848**
Stem diameter	17.593***	6.475***	1.849
Stem biomass	24.517***	21.495***	2.548
Leaf biomass	2.931	23.737***	0.377
Root biomass	16.284***	15.926***	0.317
Total biomass	27.790***	17.702***	1.323
Stem mass ratio	0.262	113.489***	0.259
Leaf mass ratio	1.961	37.634***	0.633
Root mass ratio	1.434	34.913***	0.412

## Discussion

Plants need suitable water conditions for their propagation and growth. The majority of plant species under water deficiency conditions experience retarded growth and the gradual deterioration of their morphological and physiological properties^25^. The slow gas diffusion that occurs underwater dramatically reduces oxygen and carbon dioxide influxes into plant tissues that exhibit normal respiration and photosynthesis, thus retarding plant growth^26^. Complete submergence imposes considerable stress on plant functional traits, predominantly due to oxygen deprivation, and it rapidly results in the loss of biomass and ultimately the death of many plant species^27^. *A. philoxeroides* is highly plastic and grows rapidly in both terrestrial and aquatic habitats, being recognized as a plant that tolerates waterlogging^3,4^. It may have some strategies to deal with the negative effects of water stress.

The survival rate is an important index to judge the ability of plants to withstand water stress^28^. A previous study found that *A. philoxeroides* could survive in 2 m of water for 60 days^29^. The fragments with leaves had more resource reserves than those without leaves^23^, and thus, the survival was improved in treatments with leaves, especially under submergence conditions in both of our experiments. Therefore, the ability of plants to withstand water stress is largely related to the level of plant resource reserves^30,31^.

The resource reserves level of a plant is critical to determine its tolerance to long-term submergence^31–33^. Under submergence, the main stress that plants suffer from is the decrease in oxygen supply, causing the plants to switch from aerobic respiration to anaerobic respiration, thus reducing the utilization efficiency of carbohydrates and aggravating the consumption of nutrients^28^. To alleviate the damage caused to plants by flooding, different flood-tolerant plants can adopt different strategies to adapt to flooded environments. To restore the contact between leaves and air above the water surface, some plants can accelerate the elongation of branches and leaves to escape flooding^32,34–37^. Therefore, shoot elongation is a type of plant response to submergence and is an escape trait to adapt to flooding conditions^27,38–40^. In Exp. 2, the stem length of the plant under submergence was more than 5 cm (the length before water control). Thus, the stem length was increased under submergence after water control, and the escape strategy was adopted by *A. philoxeroides* at this time^15^. However, the increase of the stem length in the treatments with leaves was more than that in the treatments without leaves, indicating that the treatments with leaves had more resource reserves, and thus, they had more advantages in the escape process (shoot elongation). In the treatments without leaves, the increase of the stem length at the 5 cm depth was more than that at the 10 cm depth. It was because when the depth of submergence was shallow, the stem reached the water surface more quickly; meanwhile, when the depth of submergence was large, the stem needed more time to reach the water surface and consumed more resource reserves, leading to a shorter stem length than that in shallow water.

If the plant cannot resume contact with the air, the plant's carbohydrates are severely depleted, and when its resource reserves are depleted, the plant may die^31,35,36,41^. Another strategy for adapting to submergence is a quiescence strategy that does not elongate the stem rapidly^40,41^. Plants with a quiescence strategy reduce consumption by limiting growth and rely on resource reserves to survive flooding^35,42–44^. In Exp. 1, the stem in treatments without leaves under submergence did not grow above the water surface after three months of water control (the stem lengths were both smaller than 5 cm at submergence depths of 5 cm and 10 cm), but the plants were still alive; thus, they adopted the quiescence strategy. Compared with the treatment with leaves, which were also submerged but the stem length exceeded the water surface (at the 5 cm depth) or reached the water surface (at the 10 cm depth), the deficiency of resource reserves caused by the lack of leaves in treatments without leaves was one of the main reasons why the stem length could not reach the water surface and a quiescence strategy was adopted under submergence. Plant growth retardation under drought conditions was also a quiescence strategy to cope with drought conditions.

The stem of *A. philoxeroides* can grow in the following two ways: one way is by increasing the number of nodes (the number of leaves is increased at the same time), and another way is by extending the internode length. Underwater, photosynthesis is a straightforward way to reduce the shortages of both oxygen and carbohydrates, alleviating stress under flooding conditions, and flood-tolerant species generally continue to develop new leaves to increase survival during submergence^27^. The development of new leaves of *A. philoxeroides* was also observed under drought and submergence, as in previous studies^15,29^. However, compared with the 97% water content, drought and submergence reduced the number of new nodes and more significantly reduced the number of new leaves. In addition, it was observed that the phenomenon of defoliation was very serious under submergence. If a plant under submergence adopts an escape strategy and wants to elongate its stem rapidly, it needs to balance the energy allocation between distributing the existing energy to the leaves for photosynthesis and distributing it to the stem for rapid elongation. The large reduction of leaves indicated that during submergence, *A. philoxeroides* allotted more energy to the stem for elongating internode growth^17^. If a plant under submergence adopts a quiescence strategy, it is necessary to reduce the energy consumption of leaves. Therefore, leaf shedding under submergence is a manifestation of the quiescence strategy in response to flooding when the resource reserves is insufficient.

To adapt to aquatic habitats, the diameter and structure of the *A. philoxeroides* stem also changes^13^. Previous studies have found that, compared with that of the terrestrial population, the stem is thicker and more hollow in the aquatic *A. philoxeroides* population, whose structures have greater buoyancy and are more suitable for aquatic habitats^8,45^. In our two experiments, we found that drought and submergence conditions decreased the stem diameter, and the stems in treatments without leaves were significantly thinner than those in treatments with leaves. This suggested that the stem diameter is also related to the plant resource reserves, and water stress is unfavourable for stem thickening.

Drought and submergence largely decreases the biomass production of wetland plants^46,47^. Compared to the 97% water content, drought and submergence significantly decreased the stem, leaf and total biomass, which is consistent with previous srudies^15,42^. Studies have shown that when *A. philoxeroides* grows in the terrestrial habitat, it develops thickened and storage roots^48,49^. Therefore, in Exp. 2, the root biomass was significantly higher under the 70% water content than under the other water conditions. However, the 70% water content was not the best environment for the fragment of *A. philoxeroides* to root and sprout, and thus, in Exp. 1 (water control with stolon fragments), the root biomass was lower under the 70% water content than under the 97% water content. Under submergence, gas diffusion and the physical status of soils were changed, which often directly affected the roots or other underground organs^50,51^. To maintain material storage, plants reduced their respiratory consumption by reducing their root biomass^52^. The root biomass decreased significantly when the plants were submerged (Exp. 2), while root growth was almost completely inhibited when the stolon fragments were submerged (Exp. 1).

To successfully capture resources in an unfavourable environment, plants often invest more in the biomass of some components than under normal conditions^53,54^. For example, to obtain O_2_, CO_2_ and light resources, the proportion of underground biomass of the erect submersible plants *Potamogeton maackianus* and *Potamogeton malaianus* decreased with increasing water level, and thus, the proportion of stem biomass increased^55^. The biomass allocation strategy of *A. philoxeroides* was similar under varying water conditions; with the increase of the water level, the stem mass ratio increased, while the leaf mass ratio and root mass ratio decreased. This was related to the elongation of the stem under the condition of more water, the leaf shedding under submergence, and the need to develop roots to absorb water under water deficit conditions. This was the manifestation of the strategy adopted by the *A. philoxeroides* to adapt to changes in the water conditions and improve the survival ability. The leaf mass ratio in the treatment without leaves was higher than that in the treatment with leaves, and it was especially significant in Exp. 2. This indicated that the plant in the treatment without leaves will greatly increase the material input ratio of leaves to further improve the photosynthetic and nutrient production capacity.

Under the condition of sufficient water, plants will constantly absorb water to replace the water taken away by transpiration and maintain normal growth. However, they will be affected by transpiration of leaves under the condition of less water. In Exp. 1, the survival rate in treatment with leaves was lower than that in treatment without leaves only at the 70% water content, but the difference was not significant. It was suggested that transpiration of leaves would reduce the reproduction of horizontally placed stolon fragments under drought condition, but not to a significant extent. The morphological characteristics and biomass accumulation of plants in the treatment with leaves were higher than those in the treatment without leaves. Therefore, the nutrients stored in the leaves could promote the vegetative reproduction of the stolon fragments and later plant growth, and they were especially beneficial for plant tolerance to submergence.

Therefore, *A. philoxeroides* has a wide ecological range and can tolerate short-term flooding and drought. But the conditions of water deficiency and submergence retarded the vegetative reproduction and growth of *A. philoxeroides* plant, and deteriorated the morphological properties and biomass accumulation. The vegetative reproduction of fragments was low under drought and submergence, and the sprouting of fragments was very unusual under 30% water content. *A. philoxeroides* could survive from the 30% soil water content to a 10 cm depth of submergence for three months, had strong vegetative reproduction and growth abilities and was most invasive under the 97% water content (wet habitats). Under adverse submergence conditions, plants can adopt different strategies, in particular, escape or quiescence strategies, depending on the plant’s own resource reserves. When there were enough resources to store, plants adopted the escape strategy under submergence, while when there were not enough resources to store, plants adopted the quiescence strategy to endure submergence conditions. The leaves on the stolon fragments increased the material resources needed for sprouting and growth. Their transpiration had effect on the reproduction of single node horizontal stolon fragments under drought condition. The response strategy could be adjusted according to the environmental conditions and the resource reserves by themselves, and thus, *A. philoxeroides* can successfully invade a variety of habitats and is widely distributed.

## Materials and methods

### Ethics statement

The experimental plants were obtained from a greenhouse at Guizhou Education University, so no permission was requested for collection. The experimental conditions were established by the research team, so no special permission was requested for the experiment. The experiment did not involve any endangered or protected species, so no specific permissions were required for the collection of plants.

## Experimental materials

*A. philoxeroides* plants were collected from Guiyang city suburb, Guizhou Province in southwest China in the middle of June 2017 and propagated vegetatively in the greenhouse of Guizhou Education University. In late July 2017, clonal fragments of similar diameters were severed from the mature, creeping stems of stock plants for use in the experiments. Each fragment was 6 cm in length and contained a single node in the middle (3 cm length internodes on the two sides of the node). There were two leaf treatments, including fragments with two leaves and fragments without leaves.

## Experimental design

**Figure 3:**
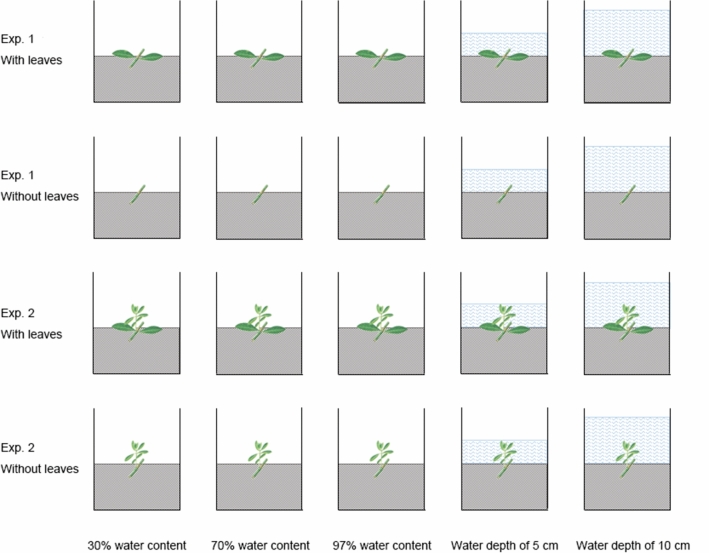
Schematic representation of the experimental design.

The experiments were conducted in a greenhouse at Guizhou Education University. A 10 cm soil depth in plastic boxes (56 cm × 41 cm × 22 cm; length × width × height) was used for the experiments. The soil was collected from Guiyang city suburbs. Stolon fragments were horizontally embedded on the soil surface with a small stone. Each box was planted with 10 stolon fragments. During the experiments, the boxes were randomly repositioned to avoid the effects of environmental conditions within the greenhouse. There were five water level conditions in each experiment: I, 30 ± 3% soil water content (simulated drought terrestrial habitat, rehydrated approximately once a week); II, 70 ± 3% soil water content (simulated terrestrial habitat with moderate water, rehydrated every 3–4 days); III, 97 ± 3% soil water content (simulated wet terrestrial habitat, rehydrated daily); IV, water depth of 5 cm (simulated submergence, rehydrated daily); V, water depth of 10 cm (simulated submergence, rehydrated daily) (Fig. 3). During the experiment, the average air temperature was 27 °C, and the average relative air humidity was 77% in Guiyang^56^. Hygrometers were used for measurement of the soil water content under the water control treatments (ZD-06, ZD Instrument Company, China).

The study consisted of two experiments. The first experiment (Exp. 1) was a water control with vegetative reproduction stolon fragments. The experiments started on 28 July 2017 and ended 3 months later on 29 October. The aim was to investigate the effects of different water conditions on sprouting, survival and later growth of fragments of *A. philoxeroides*. Fragments with and without leaves were placed on the surfaces of the soil in the plastic boxes, and the water content was controlled. There were 3 replicates of boxes for each treatment. The second experiment (Exp. 2) was the water control with plants. The experiments started on 24 July 2017 and ended 3 months later on 27 October. The aim was to investigate the effects of different water conditions on the survival and growth of *A. philoxeroides* plants. Fragments with and without leaves were placed on the surfaces of the soil in the plastic boxes and subjected to normal field water management. When the seedlings sprouted and grew to approximately 5 cm (not more than 5 cm), water control was started (on August 9). There were 6 replicates of boxes for each treatment. Therefore, there were two experiments, each with two leaf treatments and five water conditions.

## Measurements and analyses

We observed germination every day. At the end of the experiments, we harvested and counted the number of nodes and leaves and measured the stem length and stem diameter. Then, we divided each plant into roots, stems and leaves. All parts were then dried at 75 °C for 72 h and weighed. For analyses, we calculated the survival rate (the plants that sprouted all survived until the end of the experiment for Exp. 1, reproduction rate = survival rate; before the water control, all the fragments sprouted in Exp. 2) in each box. The stem mass ratio, leaf mass ratio and root mass ratio were calculated as the ratios between the biomasses of the stems, leaves, and roots, and the total biomass, respectively. The mean values of the variables measured in each box were used in the analyses.

Two-way ANOVA was used to investigate the effects of the water condition and leaf treatments on the parameters mentioned above. If significant effects were detected, then the least significant difference (LSD) (for more than three treatments) test was used to compare the means between the treatments. Prior to ANOVA, all data were checked for normality and homoscedasticity. All statistical analyses were carried out with SPSS 20.0 (Chicago, IL, USA). The differences were considered significant if* P* < 0.05.
